# Development and Validation of a New Methodology to Assess the Vineyard Water Status by On-the-Go Near Infrared Spectroscopy

**DOI:** 10.3389/fpls.2018.00059

**Published:** 2018-01-30

**Authors:** Maria P. Diago, Juan Fernández-Novales, Salvador Gutiérrez, Miguel Marañón, Javier Tardaguila

**Affiliations:** Instituto de Ciencias de la Vid y del Vino - CSIC, University of La Rioja and Gobierno de La Rioja, Logroño, Spain

**Keywords:** grapevine, water stress, stem water potential, non-invasive proximal sensing, PLS

## Abstract

Assessing water status and optimizing irrigation is of utmost importance in most winegrowing countries, as the grapevine vegetative growth, yield, and grape quality can be impaired under certain water stress situations. Conventional plant-based methods for water status monitoring are either destructive or time and labor demanding, therefore unsuited to detect the spatial variation of moisten content within a vineyard plot. In this context, this work aims at the development and comprehensive validation of a novel, non-destructive methodology to assess the vineyard water status distribution using on-the-go, contactless, near infrared (NIR) spectroscopy. Likewise, plant water status prediction models were built and intensely validated using the stem water potential (ψ_s_) as gold standard. Predictive models were developed making use of a vast number of measurements, acquired on 15 dates with diverse environmental conditions, at two different spatial scales, on both sides of vertical shoot positioned canopies, over two consecutive seasons. Different cross-validation strategies were also tested and compared. Predictive models built from east-acquired spectra yielded the best performance indicators in both seasons, with determination coefficient of prediction (${\text{R}}_{P}^{2}$) ranging from 0.68 to 0.85, and sensitivity (expressed as prediction root mean square error) between 0.131 and 0.190 MPa, regardless the spatial scale. These predictive models were implemented to map the spatial variability of the vineyard water status at two different dates, and provided useful, practical information to help delineating specific irrigation schedules. The performance and the large amount of data that this on-the-go spectral solution provides, facilitates the exploitation of this non-destructive technology to monitor and map the vineyard water status variability with high spatial and temporal resolution, in the context of precision and sustainable viticulture.

## Introduction

There is a great potential both for monitoring water stress and scheduling irrigation in commercial orchards (Fernández and Cuevas, 2010). Vineyard water status affects vegetative growth, yield, grape composition, and wine sensorial attributes (Ojeda et al., 2002; Chapman et al., 2005; Chaves et al., 2007). Assessing water status and optimizing irrigation are very interesting issues in most winegrowing countries. Of the various techniques to appraise the plant water status, plant-based methods have shown the advantage of integrating the soil and atmospheric effects (Jones, 2004). Likewise, the usefulness of different physiological parameters and their applicability for water stress detection and irrigation management in grapevines was reviewed by different authors (Jones, 2004; Acevedo-Opazo et al., 2008; Jones and Grant, 2016). However, conventional plant-based methods to monitor water stress, such as those based on the use of Scholander-type chambers, are destructive as well as time and labor consuming (Fernández, 2014). Therefore, new methods for monitoring vineyard water status are needed in sustainable water management (Fernández, 2014; Jones and Grant, 2016). In this context, novel tools have been developed for non-destructive, automated, and continuous measurements (Rodriguez-Dominguez et al., 2012; Ballester et al., 2014). Although very reliable and informative, many of these tools monitor only a single plant in the field therefore, they are unsuited to detecting spatial variation in water status within a vineyard (Baluja et al., 2012).

New technologies, sensors and computing are desirable in viticulture (Fuentes et al., 2012) to assess vineyard spatial variability. In precision viticulture the usefulness and convenience of high-spatial resolution information provided to assess plant water status zones within-vineyards was suggested by several authors (Acevedo-Opazo et al., 2010; Cohen et al., 2017). Remote sensing technologies have been applied to vineyard water status monitoring (Baluja et al., 2012; Bellvert et al., 2016). Recently, lateral and proximal sensing technologies, as thermography and near infrared (NIR) spectroscopy have been also used for on-the-go assessment of vineyard water status (Diago et al., 2017; Gutiérrez et al., 2017; Fernández-Novales et al., 2018). Still, it is necessary to take a further step and to develop reliable, fully tested solutions that make use of this kind of contactless, proximal sensing technology in the context of precision viticulture. Thus, the need of a suitable methodology for fast, on-the-go, vineyard monitoring could be considered as the next barrier to be crossed, and NIR technologies are prone to ease this step.

NIR spectroscopy is a powerful analytical technique that enables rapid and non-destructive data acquisition, easy usage and little sample preparation, which has been used for in-field measurements (Cozzolino, 2014). The NIR region is the part of the electromagnetic spectrum between 750 and 2500 nm, and it is related to molecular overtones and combinations of these fundamental vibrations due to the stretching and bending of N-H, O-H, and C-H groups. For this reason, it can be used for quantitative and qualitative analyses (Williams and Norris, 2001). The main constituent that can be found in leaves is water, so NIR spectral measurements performed upon their surface would result in high levels of reflectance linked to O-H bands, i.e., 760, 970, 1,450, and 1,940 nm (Nicolaï et al., 2007), being this spectral range potentially informative about water content and behavior. However, spectral data usually contain a wide number of variables, which range from several hundreds to thousands of them, a fact that highly difficults the discovering of direct correlations between the spectral variables with the trait that needs to be modeled. Because of this, the help of the multivariate analytical method of chemometrics is always virtually compulsory. Currently, statistical algorithms are used for the development of multivariate models that grants a fair prediction capability from a spectral input, such as NIR measurements, providing a reliable tool for building up calibration and prediction models. Also, different spectral filtering procedures and pre-processing mathematical techniques are applied to the raw spectral input to improve the prediction capability of the models (Geladi et al., 2003; Cozzolino et al., 2011; Dambergs et al., 2015).

A few studies have investigated the potential of NIR spectroscopy to enable real-time monitoring of the grapevine during the ripening process at leaf level, and also to assess a rapid quality control of plant water status (Santos and Kaye, 2009; De Bei et al., 2011; Gutiérrez et al., 2016; Tardaguila et al., 2017). These authors have shown the performance of different NIR portable manual devices in contact with grapevine leaves to determine the plant water status, either leaf (ψ_l_) or stem water potential (ψs) under field conditions. Two recent works have evaluated the capability of contactless NIR spectroscopy mounted on an all-terrain-vehicle for the estimation of grapevine stomatal conductance (g_s_) on a stop and go mode (Diago et al., 2017) and to quantify and discriminate different water regimes in a commercial vineyard (Fernández-Novales et al., 2018). These studies did confirm the availability of NIR spectral technology as a potential methodology for the replacement of classic water status indicators, suitable for a fast, on-the-go monitoring of a vineyard plot. Nevertheless, a full proposal in this direction, involving a wide testing in a real-scenario and in different seasons, seems to be desirable.

The goal of this work was to develop and validate a new, non-destructive methodology for the on-the-go assessment of the water status of a commercial vineyard making use of contactless NIR spectroscopy. A comprehensive study that involved the development of prediction models of a reliable plant water status indicator, such as the stem water potential was carried out. The NIR-based built models comprised a high number of samples acquired at the two sides of the canopy, during two different seasons, at two spatial scales, and were validated using different cross-validation approaches. Implementation of such prediction models to map the spatial variability of the vineyard water status was also aimed.

## Materials and methods

### Experimental layout

The study was conducted in a commercial Tempranillo (*Vitis vinifera* L.) vineyard (clone 776 on rootstock Richter 110) located in Tudelilla, La Rioja, Spain (Lat. 42°18′ 18.26″, Long. −2°7′ 14.15″, Alt. 515 m) over two consecutive seasons, from June to the end of September 2015 and from early July to late August 2016. Grapevines were planted in 2002 (north-south orientation) with vine spacing of 2.60 m between rows and 1.20 m between vines, and trained to a vertically shoot-positioned trellis system on a double-cordon Royat.

**Figure 1:**
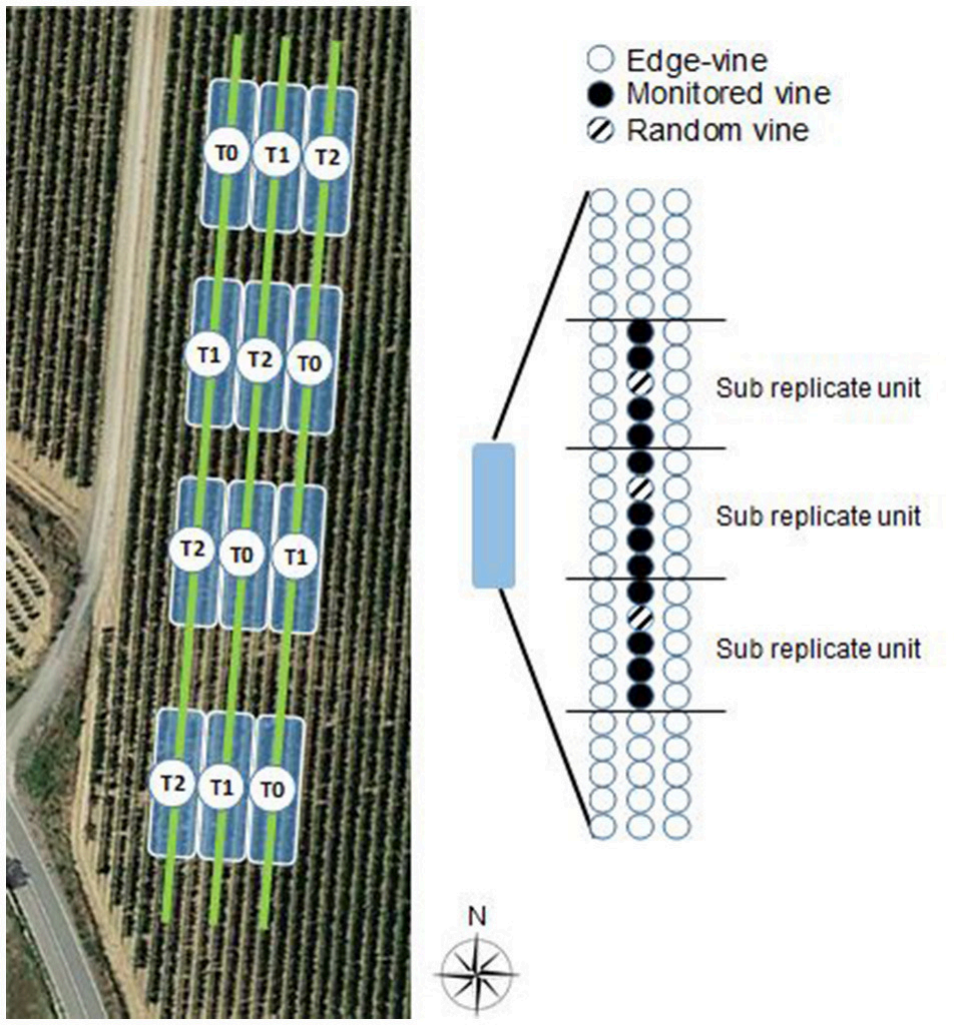
Experimental layout following a completely randomized block design with four blocks and three irrigation treatments (T0: full irrigation, T1: moderate irrigation, T2: no irrigation) established in a Tempranillo, vertically shoot positioned vineyard located in La Rioja (Spain). Close-up of a given field replicate, involving three adjacent rows, of which the middle one was monitored with the NIR spectrophotometer, and three vines per replicate were randomly selected for the measurement of the stem water potential (ψ_s_), one per each sub replicate unit.

With the aim of creating an ample variability of grapevine water status, a completely randomized block design (Hinkelmann and Kempthorne, 2007) with four blocks and three different water regimes was set (Figure 1). The three water treatments were:

- T0: Full irrigation. Two water pipelines were installed and provided a total of 406.5 mm H_2_O/m^2^ in the studied period in 2015, and 598.0 mm H_2_O/m^2^ in 2016.
- T1: Moderate irrigation. One water pipeline was installed. The total amount of delivered water in the studied period was 221.7 mm H_2_O/m^2^ in 2015 and 190.7 mm H_2_O/m^2^ in 2016.
- T2: No irrigation. No irrigation was applied during the whole experiment in any of the two seasons.

For each water regime, four replications (one per block) were set up, making up a total of 12 replications (Figure 1). Each replication comprised three adjacent rows and 25 plants in each row. Of these, only the middle row, and the 15 middle plants of the 25 vines of this middle row were considered for measurement. Each group of five vines within the 15 middle ones of each replication will be named as sub replicate unit hereafter (Figure 1). The adjacent rows and the first and last five vines per replication were not considered to avoid any edge effect. The vines subjected to the water regimes T0 and T1 were irrigated at four different equally-distanced times of the day during 30 min each, making up a total of 2 h of watering per day.

Weather data were recorded at 30 min intervals by a meteorological station property of La Rioja Government, next to the experimental vineyard. The average air temperature (T) and relative humidity (RH) were recorded at 30 min intervals in the two seasons. Additionally, for the dates and time interval at which measurements were taken (solar noon, between 14:00 and 15:30 h), the vapor pressure deficit (VPD) was calculated.

### On-the-go spectral measurements

**Figure 2:**
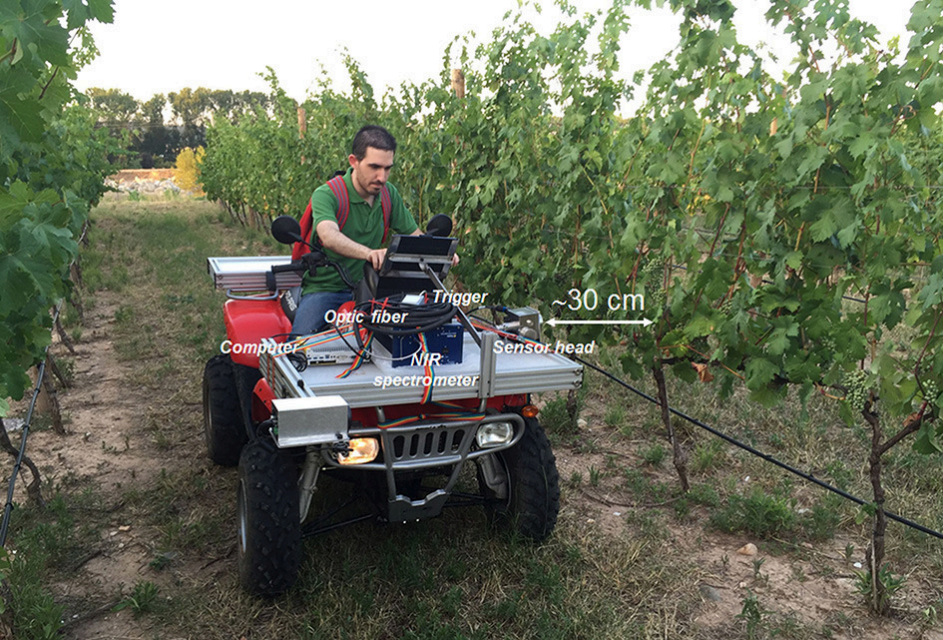
Illustration of the setup of the near infrared system operating from the moving all-terrain vehicle for vineyard water status monitoring. (The authors declare that written and informed consent has been obtained from the depicted individual in this image, for the publication of this identifiable image).

On-the-go spectral measurements in the vineyard were carried out using a NIR spectrometer (PSS 2120, Polytec GmbH, Waldbronn, Germany) which operates in the wavelength range 1100–2100 nm (4 nm resolution; 251 datapoints per spectrum). The spectrometer was an active NIR optical device with a polychromator as reflection light source selector, and Indium Gallium Arsenide (InGaAs) diode array detectors. The system includes a sensor head for light emission (by an integrated 20 W tungsten lamp) and capturing, and a processing unit, both linked by an optical fiber (Figure 2). The whole spectral system was mounted in the front part of an all-terrain-vehicle (Trail Boss 330, Polaris Industries, Minnesota, USA), aiming to the left and able to make spectral acquisitions controlled by a physical trigger while the all-terrain-vehicle is in motion. The sensor head was placed at a height of 0.95 m from the ground, to cover the mid-upper part of the grapevine's canopy (just above the fruiting zone) (Figure 2). The measurements were conducted contactless (no contact with the canopy occurred), at ~30 cm distance from the canopy. The diameter of the measurement window was 19 mm. On-the-go spectral measurements were acquired on both sides of the canopy (east and west) at an average speed of 5 km/h and rate of spectral acquisition of 24 Hz.

### Measurement of the stem water potential (ψ_s_)

Midday stem water potential (ψ_s_) was used as the reference method to assess the plant water status. For each field replication three random vines within the 15 monitored (Figure 1) were selected (one vine of the first five plants, another of the centered five plants, and the third one of the last five plants), and one adult leaf of the mid-upper part of the canopy per vine was tagged and its ψ_s_ determined. Therefore, 36 leaves were measured each day, making a total of 324 measurements of ψ_s_ in season 2015, and 216 in 2016.

Measurements of ψ_s_ were conducted at solar noon (at the same time interval as spectral acquisition) using a Schölander pressure bomb (Model 600, PMS Instruments Co., Albany, USA). Prior to the determination of ψ_s_, the tagged leaves were covered with aluminum foil and allowed to dark adaptation during 1 h.

### Spectral processing

Spectral data handling and calibration models were carried out with MATLAB (version 8.5.0, The Mathworks Inc., Natick, MA, USA). PLS Toolbox (version 8.1, Eigenvector Research, Inc., Manson, WA, USA) was used for principal component analysis (PCA) and partial least squares (PLS) regression.

Spectral processing involved several steps. The first one consisted on the allocation of the acquired spectra to the different groups of vines within each field replicate. Likewise, for each date and side of the canopy (east and west), the raw spectra corresponding to the 15 middle plants (around 360 spectra) per field replication were equally distributed in three groups (120 spectra per group): one corresponding to the first five plants (first sub replicate unit), another to the following five plants (second sub replicate unit), and the third group of spectra referred to the last five plants (third sub replicate unit). This allocation was made on the basis that the speed of the all-terrain vehicle was kept constant during spectral acquisition. Within each field replicate, for each of the three sub replicate units, the average spectrum was computed. These average spectra were then linked with their corresponding value of ψ_s_ making up a total dataset of 324 samples per canopy side in 2015, and a dataset of 216 samples in 2016.

Due to different kind of spectra collected during the measurements, including gaps, wood, metal, etc., a filtering step was needed. In order to retain only those spectra corresponding to grapevine leaves, a spectra comparison was performed using the “Spectra Comparison & Filtering” tool from the SL Utilities software (version 3.1, Polytec GmbH, Waldbronn, Germany), and providing a static, well-taken signature spectrum of a grapevine leaf for the comparisons.

The third step involved the pre-processing of the average spectra to remove the effects of light scattering and to compensate for baseline offset and bias. Several combinations of spectral pre-processing filters were tested and those yielding the best prediction outputs were finally chosen. These filters involved the use of standard normal variate (Barnes et al., 1989; Dhanoa et al., 1995) and the application of the Savitzky-Golay smoothing and derivative procedures, selecting distinct values for the window size and degree of the derivative. Derivatives were used to accentuate small bands and to resolve overlapping peaks (Savitzky and Golay, 1964).

In the fourth step, PCA was used to reduce the dimensionality of the data, to examine any possible grouping and to identify potential outliers by studying score plots using Q residuals and Hotelling's T^2^ statistic (Brereton, 2003). The Q statistic was calculated as the sum of squares of the residuals (Jackson, 2003). Equation (1) shows the Hotelling *T*^2^ computation procedure (Hotelling, 1931), where: *p* is the number of variables (PC scores considered); *n* is the number of samples; and *F* the critical value for a Fisher distribution with α confidence level.

$$\begin{array}{l} T_{p,n,\alpha }^{2}=\frac{p\left(n-1\right)}{n-1}\;F_{p,n-p,\alpha } \end{array}$$

### Chemometrics and data analysis

Calibration, validation, and prediction models of grapevine water status were built using PLS regression, where the processed spectra were the inputs and the values of ψ_s_ the reference indicator. PLS has proved to be an accurate, robust, and reliable chemometric method (Wold et al., 2001) to analyse spectral data, as it is capable to deal with a vast amount of data, especially when the number of wavelengths largely surpasses the number of samples. PLS water status models for ψ_s_ prediction were built using the 256 spectral datapoints (X matrix) and the ψ_s_ values (Y matrix) as inputs. Individual models for each season (2015 and 2016) and a global one involving all data from the two seasons were developed. Models were built at two different spatial scales: (a) considering the three ψ_s_ per field replicate individually (seasons 2015 and 2016), and (b) considering an average spectrum and ψ_s_ value per field replicate (only in 2015). In the two seasons, models were built for each side of the canopy independently (east and west) and using data from both sides. For the latter approach, a new dataset including spectral measurements from east and west sides datasets was generated. Special care was taken to make this new dataset as representative as those corresponding to a single canopy side. For that purpose, a pseudorandom sample selection of the same amount of data per canopy side, water regime, and measurement day was conducted, to end up with a new dataset with a total number of samples equal to those of the east or west sides. For the global model of the two seasons, modeling was conducted only for the east side.

With the aim of building robust models capable of predicting totally unknown samples, the original dataset of spectra was split up into two independent datasets: a calibration one (comprising 80% of all data) and an external validation set (comprising the remaining 20% of original data). The calibration dataset was used to train and to perform an internal cross-validation of the model, while the external validation set was only utilized for prediction purposes, using the calibration models. Two different methods of internal cross-validation were tested: (a) 10-fold venetian blind cross validation, and (b) leave one day out cross validation. In a *n*-fold venetian blind cross validation, each fold *i* is built taking samples from the dataset of a *n*-multiple position until the end of the dataset (samples *i, i* + *n, i* + *2n, i* + *3n*, …). Once the folds are built, a traditional *n*-fold cross validation is carried out, in which *n* models are trained with *n–1* folds, and tested with the remaining fold, rotating the latter until all of them have been used. The average performance of the *n* models is finally computed. The second internal validation approach, the leave one day out cross validation, is similar to the leave one out cross validation, in which a single observation (in this case data from one date) is used to internally validate the training model built with the remaining observations (remaining dates). This is repeated such that each observation (each date) in the original dataset is used once as the validation data. For each model, the optimal number of latent variables was selected as that yielding the minimum root mean square error of cross validation (CV-RMSE). To evaluate the quality of the best models obtained, the coefficient of determination (R^2^) and the root mean square error (RMSE) of calibration (${\text{R}}_{\text{c},}^{2}$ calibration RMSE), cross-validation (${\text{R}}_{\text{cv}}^{2}$, CV-RMSE), and prediction (${\text{R}}_{\text{p}}^{2}$, prediction RMSE) were calculated.

### Mapping

To illustrate the capability of the developed methodology to assess the vineyard water status variability, maps of the predicted values of ψ_s_ in the monitored vineyard plot were built using a multilevel b-spline interpotation with QGIS 2.18 (Free Software Foundation, Boston, MA, USA) for two dates, one of season 2015 and another one from season 2016.

## Results

### Environmental data and vineyard water status

The nine dates in 2015, and six dates in 2016, at which vineyard measurements were taken involved very different weather conditions, in terms of air T, RH, and VPD. The average values of air T, RH, and the computed VPD at the time interval of vineyard monitoring (solar noon) and ψ_s_ measurements in the two seasons are summarized in Table 1. In season 2015 the average air T during vineyard monitoring hours ranged from 20.4°C in September, to 32.0°C in the first 2 weeks of August, while the RH varied from 20.0% at mid August to 50% at mid September. The highest evapotranspiration demand occurred in August, with values closed to 3.0 kPa, while the lowest demand, which was less than half the maximum recorded value, happened during the first week of September (Table 1). In 2016, a larger range of average air T, RH, and VPD values during vineyard monitoring hours was recorded. Likewise, average air T ranged from 18.7°C (mid July) to 32.8°C (late August), RH varied from 22.5 to 53.0% and VPD fluctuated from 1.31 kPa at mid July to 3.39 kPa at the third week of August.

**Table 1:** Average values of air temperature (T), relative humidity (RH), and vapor pressure deficit (VPD) at the time of measurement (solar noon, between 14:00 and 15:30 h, GMT+1 local time) at the vineyard site for the dates of monitoring in season 2015 and 2016.

**Variable**					**Date of measurement**				
	**Season 2015**								
	**23rd Jul**	**28th Jul**	**6th Aug**	**12th Aug**	**19th Aug**	**26th Aug**	**7th Sep**	**11th Sep**	**18th Sep**
Average air T (°C)	29.2	28.2	31.6	32.0	26.9	31.1	20.4	25.4	20.4
RH (%)	44.0	35.0	37.5	36.5	20.0	33.5	42.0	50.0	39.0
VPD (kPa)	2.24	2.46	2.97	3.02	2.85	2.99	1.36	1.59	1.43
	**Season 2016**								
	**7th Jul**	**13th Jul**	**20th Jul**	**28th Jul**	**11th Aug**	**23rd Aug**	–	–	–
Average air T (°C)	27.2	18.7	29.1	29.2	22.6	32.8	–	–	–
RH (%)	53.0	48.5	40.5	22.5	38.0	32.5	–	–	–
VPD (kPa)	1.67	1.31	2.38	3.10	1.74	3.39	–	–	–

*Jul, July; Aug, August; Sep, September*.

**Figure 3:**
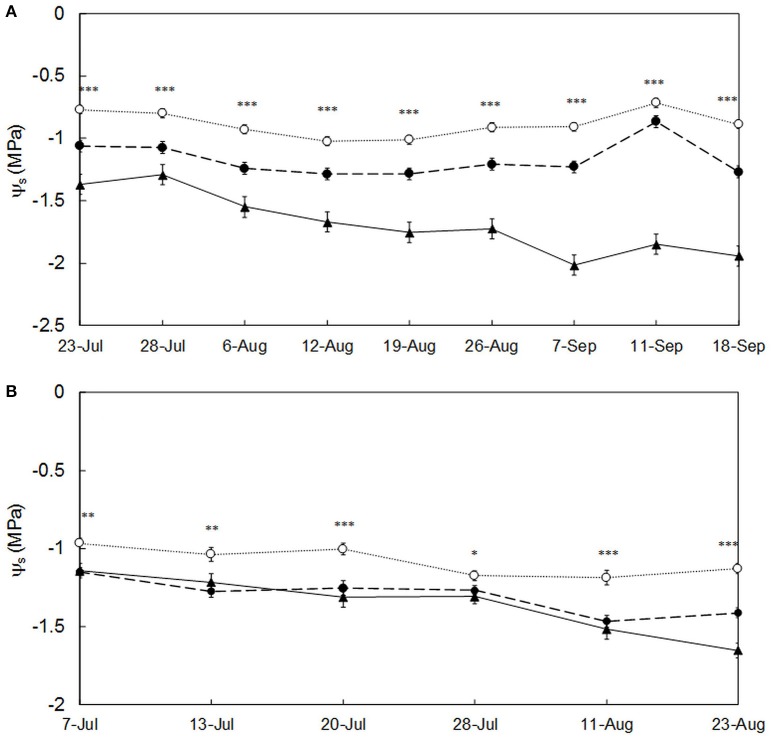
Evolution of the stem water potential (ψ_s_) for each irrigation treatment (T0: full irrigation, T1: moderate irrigation, T2: no irrigation) across the ripening season in **(A)** 2015 and **(B)** 2016. For each date, the averaged data (*n* = 12) for each irrigation treatment was represented. Error bars correspond to the standard error. Significant differences among the three irrigation treatments at ^*^p < 0.05, ^**^p < 0.01, or ^***^p < 0.001 were observed at all dates. (T0 is represented by white dots and dotted line; T1 is represented by black dots and dashed line; T2 is represented by black triangles and solid line).

The imposed irrigation treatments successfully generated an ample plant water status variability within the vineyard (Table 2), and led to significant differences (*p* < 0.05) in ψ_s_ among them across the different measuring dates in both seasons (Figure 3). Considering the individual measurements of grapevine ψ_s_, these ranged from −0.55 MPa (no water stress) to −2.25 MPa (severe water stress) in 2015, and from −0.75 MPa (no water stress) to −1.95 MPa (severe water stress) in 2016 (Van Leeuwen et al., 2009). At field replication (only for season 2015) level, in which the three individual ψ_s_ measurements per replication were averaged, the ψ_s_ ranged from −0.71 MPa (no water stress) to −2.02 MPa (severe water stress) (Table 2).

**Table 2:** Descriptive statistics of the stem water potential (ψ_s_) data measured across the dates of the whole experiment in seasons 2015 and 2016, expressed in MPa.

**Irrigation treatment**				**Stem water potential (ψ_s_)**				
	**Season 2015**							
	**Field replication (*n* = 108)**				**Grapevine (*n* = 324)**			
	**Min**.	**Max**.	**Mean**	**SD**	**Min**.	**Max**.	**Mean**	**SD**
T0-Full irrigation	−1.02	−0.71	−0.88	0.105	−1.35	−0.55	−0.85	0.161
T1-Moderate irrigation	−1.29	−0.87	−1.17	0.141	−1.65	−0.65	−1.16	0.235
T2-No irrigation	−2.02	−1.29	−1.69	0.245	−2.25	−1.10	−1.67	0.284
**Irrigation treatment**	**Season 2016**							
					**Grapevine (*n* = 216)**			
					**Min**.	**Max**.	**Mean**	**SD**
T0-Full irrigation	**- - -**				−1.45	−0.75	−1.08	0.151
T1-Moderate irrigation					−1.70	−1.00	−1.30	0.160
T2-No irrigation					−1.95	−0.85	−1.36	0.254

*Results are shown by vine and averaged by field replicate (an average ψ_s_ value was computed from the three individual measurements) for season 2015 and by vine for season 2016.Min, minimum; Max, maximum; SD, standard deviation*.

In 2015, as the season progressed, the ψ_s_ of plants subjected to irrigation (T0 and T1) slightly decreased until the 12th of August and remained constant or even increased from the 26th August onwards, to drop again in the last week of measurement (Figure 3A). The recovery of the ψ_s_ values in irrigated plants from the end of August was caused by the lower evaporative demand (lower VPD, Table 1) and some rains occurring at the beginning of September (data not shown). In the case of non-irrigated vines (T2) their water status steadily diminished (more negative values of ψ_s_) until the 7th of September, and slightly recovered also after the September rains (Figure 3A). In 2016, a slight decreasing trend of ψ_s_ was observed for all treatments until the 28th of July (Figure 3B). From then onwards the ψ_s_ remained mostly constant for T0 and became more negative for T1 and T2. The differences in ψ_s_ for T1 and T2 in season 2016 (Figure 3B) were much less marked than those from season 2015 (Figure 3A).

### Spectral measurements and regression models for grapevine water status assessment

**Figure 4:**
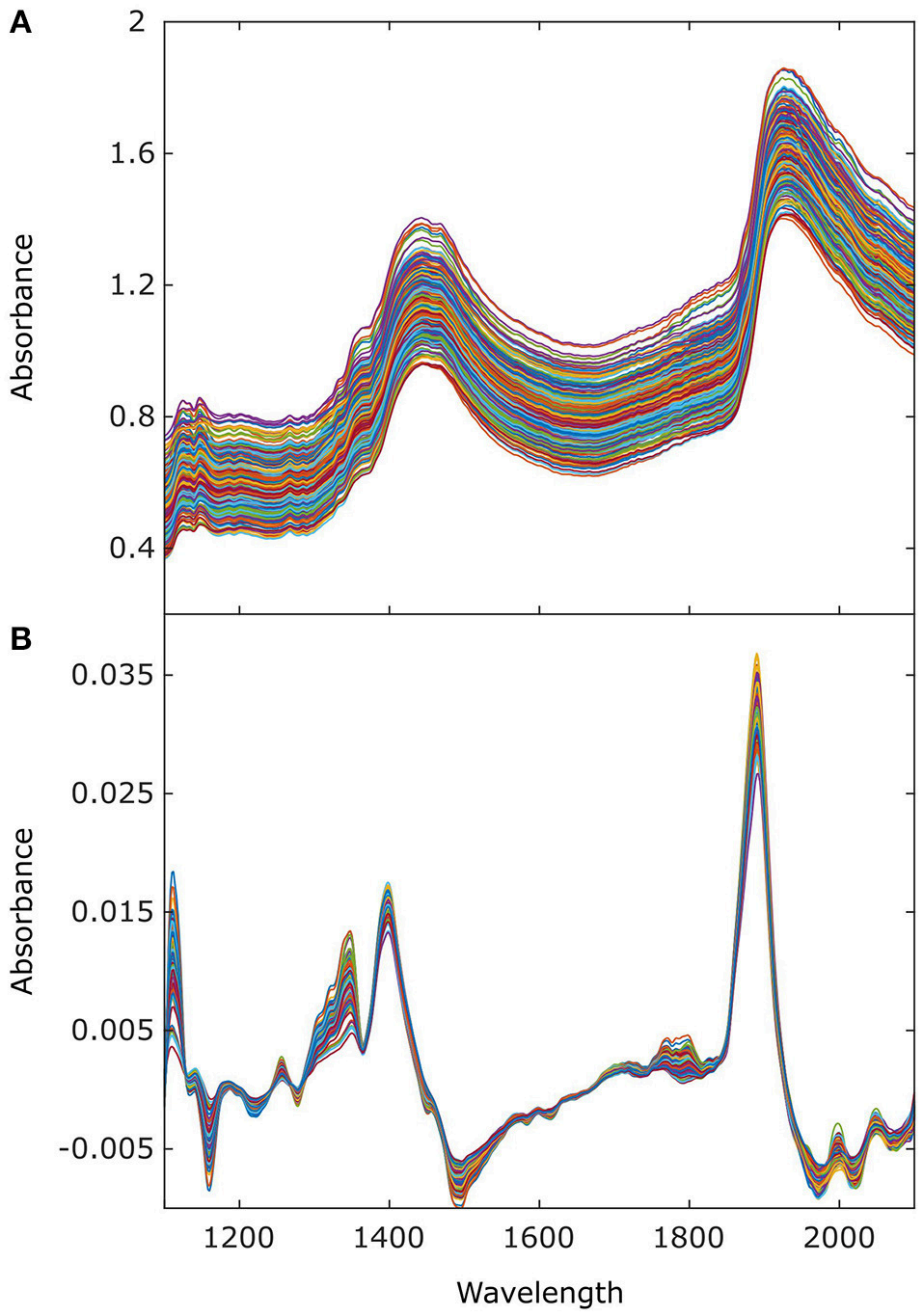
Absorbance **(A)** raw, and **(B)** first derivative spectra acquired on-the-go (at 5 km/h) in the vineyard, on the east side of the canopy along nine dates from July to September 2015.

The absorbance spectra of the grapevine canopies in the wavelength range of study (1,100–2,100 nm) (Figure 4A) and their first-derivative signal (Figure 4B) clearly revealed two absorption peaks, at ~1,450 nm, which corresponded to the first overtone of the symmetric and asymmetric hydroxyl (OH) bond stretching and/or combination bands, and around 1,940 nm, which can be assigned to the combination of the OH stretching and bending bands. Stretching, bending, and combinations are vibrational reactions of the organic groups to the electromagnetic excitation induced by NIR spectroscopy. Since leaves are mostly constituted by water, the prevalence of the OH group absorbance in their NIR spectra is well-justified (Nicolaï et al., 2007).

Table 3 summarizes the best regression models of ψ_s_ obtained for each canopy side and the two sides, at the two scale levels: at field replication scale (only for season 2015), and at a smaller, sub replication unit scale, for both seasons (2015 and 2016). Diverse pre-processing operations were applied for east, west and the two-side models, but all of them involved the Savitzky-Golay first derivative, although the size of the window (7 vs. 15) varied among the models. Following the Residuals (Q) and Hotelling values (*T*^2^) 9.9% of the in-field acquired spectra were considered samples with atypical spectra and removed in 2015, and 3.7% in 2016. Likewise, in season 2015, of the 324 sub replicate unit spectra, 32 were discarded, while from the 108 field replicate spectra, only four were removed. In 2016 only eight samples were discarded. The number of latent variables to build the models was eight in all cases in 2015, and nine in 2016 and the global approach (2015 and 2016). In general, the models built at the field replicate scale (season 2015) showed better performance indicators (larger values of R^2^ and smaller RMSE) than those generated from the sub replicate units' data (Table 3). In the two seasons, the best models were obtained using the spectra acquired from the east side, with calibration and cross validation R^2^ values ranging from 0.79 to 0.90, and 0.71 to 0.83 respectively, and calibration RMSE and CV-RMSE below 0.173 and 0.203 MPa, respectively in 2015, and 0.103 and 0.119 MPa in 2016. Similarly, a noteworthy performance was also observed for the prediction models (external validation), with ${\text{R}}_{\text{p}}^{2}$ above 0.85 and prediction RMSE around 0.150 MPa for the two modeling scales in 2015, and ${\text{R}}_{\text{p}}^{2}$ equal to 0.68 and prediction RMSE of 0.132 MPa in 2016. The performance of the models derived from the east & west dataset, comprising spectral data from the two sides of the canopy, was also remarkable, with R^2^ and RMSE values that lied within those of the individual, east and west models (Table 3).

**Table 3:** Calibration and validation statistics of the best models obtained to predict the midday stem water potential (ψ_s_) in grapevines under field conditions from on-the-go NIR spectroscopy at the sub-replicate unit, and field replicate scales.

**Modeling variables and scale**			**Calibration^a^**		**Cross validation^a^**				**Prediction^c^**	
					**10-fold**		**LODO^b^**			
**Season**	**Canopy side**	**Spectral treatment**	**RMSE**	**${\text{R}}_{\text{c}}^{2}$**	**CV-RMSE**	**${\text{R}}_{\text{c}\text{v}}^{2}$**	**CV-RMSE**	**${\text{R}}_{\text{c}\text{v}}^{2}$**	**RMSE**	**${\text{R}}_{\text{p}}^{2}$**
	**Sub Replicate Unit**									
	East	SNV+D1W15	0.156	0.86	0.171	0.83	0.192	0.77	0.151	0.86
	West	SNV+D1W15	0.195	0.78	0.214	0.73	0.251	0.71	0.188	0.78
	East & West	SNV+D1W7	0.168	0.83	0.190	0.79	0.253	0.72	0.173	0.81
	**Field Replicate**									
2015	East	D1W15	0.173	0.90	0.171	0.82	0.203	0.79	0.150	0.85
	West	SNV+D1W15	0.160	0.85	0.207	0.74	0.222	0.82	0.194	0.74
	East & West	D1W7	0.132	0.89	0.189	0.79	0.230	0.81	0.167	0.84
	**Sub Replicate Unit**									
2016	East	SNV+D1W15	0.103	0.79	0.119	0.71	–	–	0.132	0.68
	West	D1W7	0.111	0.77	0.131	0.68	–	–	0.131	0.54
	East & West	D1W7	0.106	0.78	0.128	0.68	–	–	0.133	0.62
	**Sub Replicate Unit**									
2015 & 2016	East	SNV+D1W15	0.178	0.74	0.187	0.71	0.227	0.59	0.191	0.69

a *Number of samples (n) used for the development of calibration and cross validation (10-fold) models. Season 2015: 234 for East and East & West, and 238 for West models at the sub replicate unit scale. At the field replicate level, 84 data were used for East and 86 for West, and East & West models. Season 2016: 165 samples for East, West and East &West models. Seasons 2015 & 2016: 384 samples*.

b *Number of samples (n) used for the development of cross validation models using the LODO approach. Season 2015: 318 for East and East and East & West, and 324 for West models at the sub replicate unit scale. At the field replicate level, 102 data were used for East and 104 for West, and East & West models. Seasons 2015 & 2016: 496 samples*.

c *Number of samples (n) used for prediction or external validation. Season 2015: 54 for all canopy side models at the sub replicate unit scale, and 18 at the field replicate level. Season 2016: 43 samples for East, West and East &West models. Seasons 2015 & 2016: 97 samples*.

*SNV, standard normal variate; DnWm, Savitzky-Golay filter with n-degree derivative, window size of m; RMSE, root mean square error (MPa); ${\text{R}}_{\text{c}}^{2}$, determination coefficient of calibration; CV-RMSE, root mean square error of cross-validation (MPa); ${\text{R}}_{\text{cv}}^{2}$, determination coefficient of cross-validation; ${\text{R}}_{\text{p}}^{2}$, determination coefficient of prediction; 10-fold, 10-fold venetian blind cross validation; LODO, leave one day out cross validation*.

**Figure 5:**
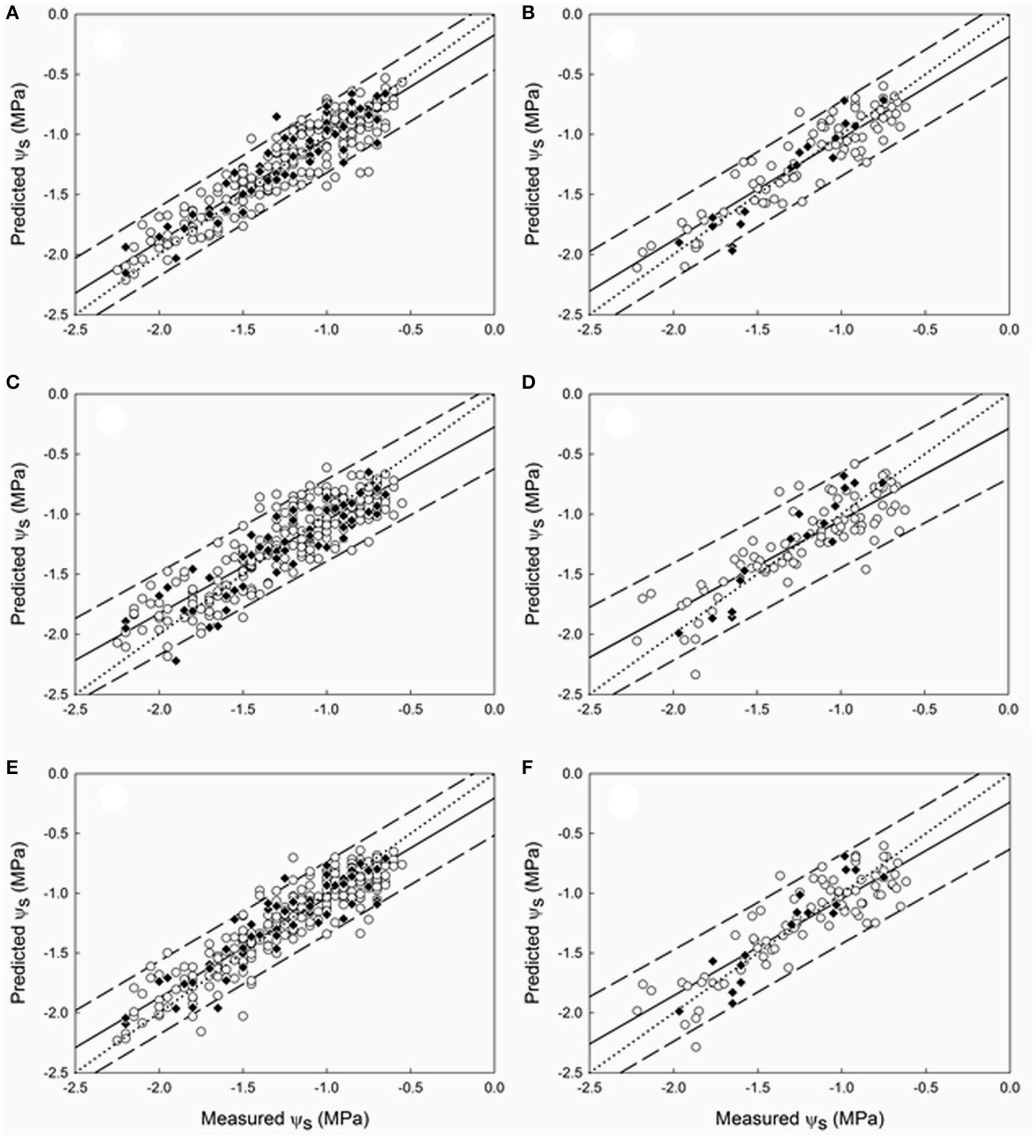
Regression plots of ψ_s_ estimation using the best PLS models developed from data of season 2015 at the sub replication unit scale **(A,C,E)** for **(A)** east (${\text{R}}_{\text{p}}^{2}$ = 0.86; Prediction RMSE = 0.15 MPa), **(C)** west (${\text{R}}_{\text{p}}^{2}$ = 0.78; Prediction RMSE = 0.19 MPa), and **(E)** east & west (${\text{R}}_{\text{p}}^{2}$ = 0.81; Prediction RMSE = 0.17 MPa) sides of the canopy. At the field replication scale **(B,D,F)** for **(B)** east (${\text{R}}_{\text{p}}^{2}$ = 0.90; Prediction RMSE = 0.14 MPa), **(D)** west (${\text{R}}_{\text{p}}^{2}$ = 0.74; Prediction RMSE = 0.19 MPa), and **(F)** east & west (${\text{R}}_{\text{p}}^{2}$ = 0.84; Prediction RMSE = 0.17 MPa) sides of the canopy. (○) 10-fold cross validation; (♦) prediction. Solid line represents the regression line and dotted line refers to the 1:1 line. Prediction confidence bands are shown at a 95% level (dashed lines).

**Figure 6:**
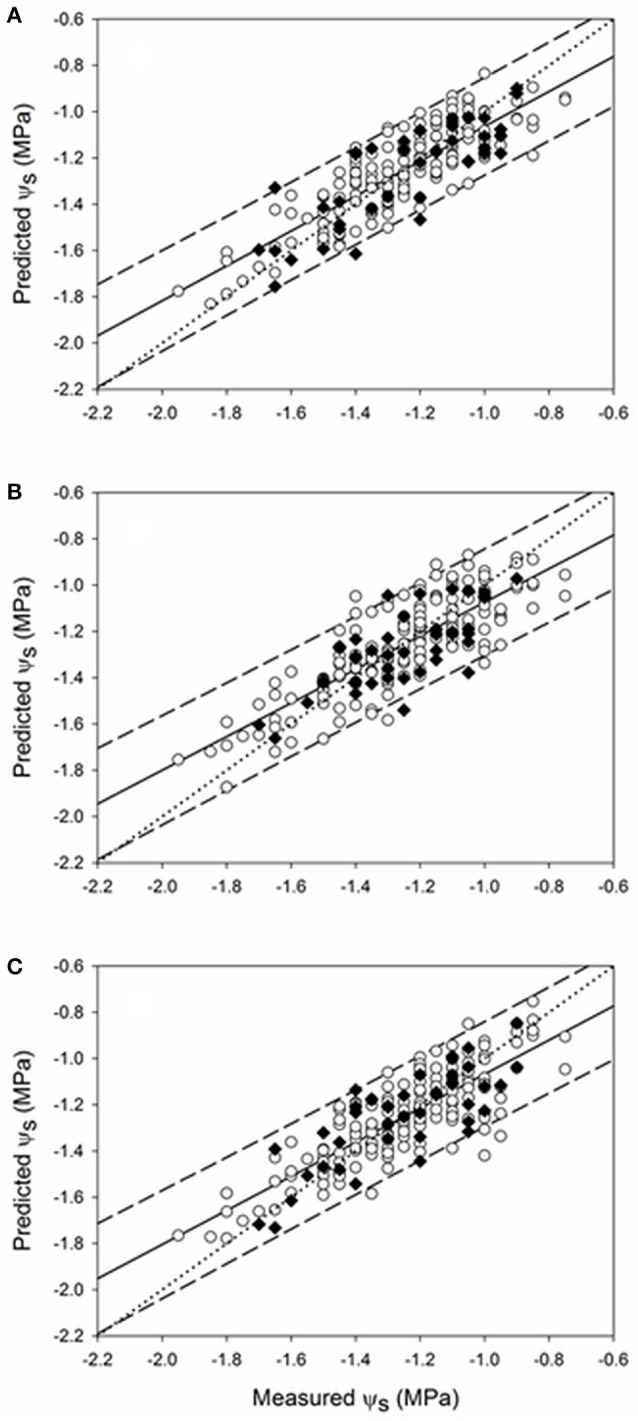
Regression plots of ψ_s_ estimation using the best PLS models developed from data of season 2016 at the sub replication unit scale for **(A)** east (${\text{R}}_{\text{p}}^{2}$ = 0.68; Prediction RMSE = 0.132 MPa), **(B)** west (${\text{R}}_{\text{p}}^{2}$ = 0.54; Prediction RMSE = 0.131 MPa), and **(C)** east & west (${\text{R}}_{\text{p}}^{2}$ = 0.62; Prediction RMSE = 0.133 MPa) sides of the canopy. (○) 10-fold cross validation; (♦) prediction. Solid line represents the regression line and dotted line refers to the 1:1 line. Prediction confidence bands are shown at a 95% level (dashed lines).

The regression plots for the best prediction models for ψ_s_ corresponding to seasons 2015 and 2016 are shown in Figures 5, 6, respectively. In 2015, a wide data range was covered by the samples, from −2.20 to −0.60 MPa. All samples from the ψ_s_ models [east (Figures 5A,B), west (Figures 5C,D), and east & west (Figures 5E,F)] exhibited a very good fit along the correlation lines and were mostly within the 95% confidence bands. In season 2016 (Figure 6), the range of ψ_s_ (from −1.95 to −0.75 MPa) was shorter than that of 2015 but similarly to the previous year, samples mostly lied within the 95% prediction confidence intervals.

**Figure 7:**
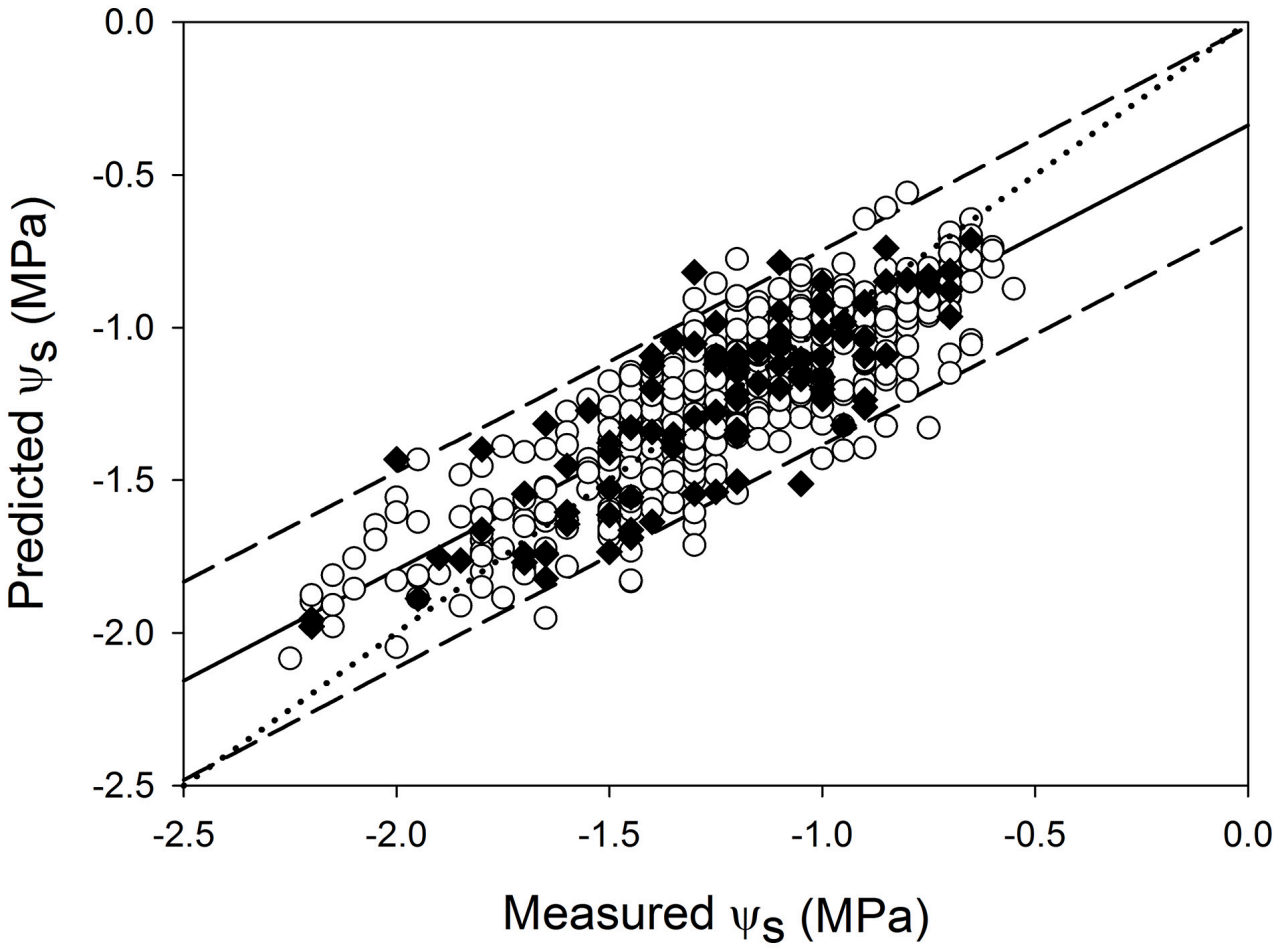
Regression plots of ψ_s_ estimation using the best PLS model developed from data of seasons 2015 and 2016 at the sub replication unit scale for the east side of the canopy (${\text{R}}_{\text{p}}^{2}$ = 0.69; Prediction RMSE = 0.191 MPa (○) 10-fold cross validation; (♦) prediction. Solid line represents the regression line and dotted line refers to the 1:1 line. Prediction confidence bands are shown at a 95% level (dashed lines).

The global model, involving data from the two seasons (2015 and 2016), was only built for the east side, as it was the one yielding the best performance results in the individual models for each year. Values of R^2^ ~ 0.70 and RMSE ~0.190 MPa were obtained for calibration, cross validation (10-fold) and prediction using the 15 measuring dates of the two seasons altogether (Table 3). For the best prediction global model (Figure 7), samples fitted along the correlation line and mostly lied within the 95% confidence intervals.

The two cross validation methods tested yielded similar results, although improved performance in terms of CV-RMSE (lower values) were obtained for the 10-fold venetian blind approach (Table 3).

### Mapping of the vineyard water status

**Figure 8:**
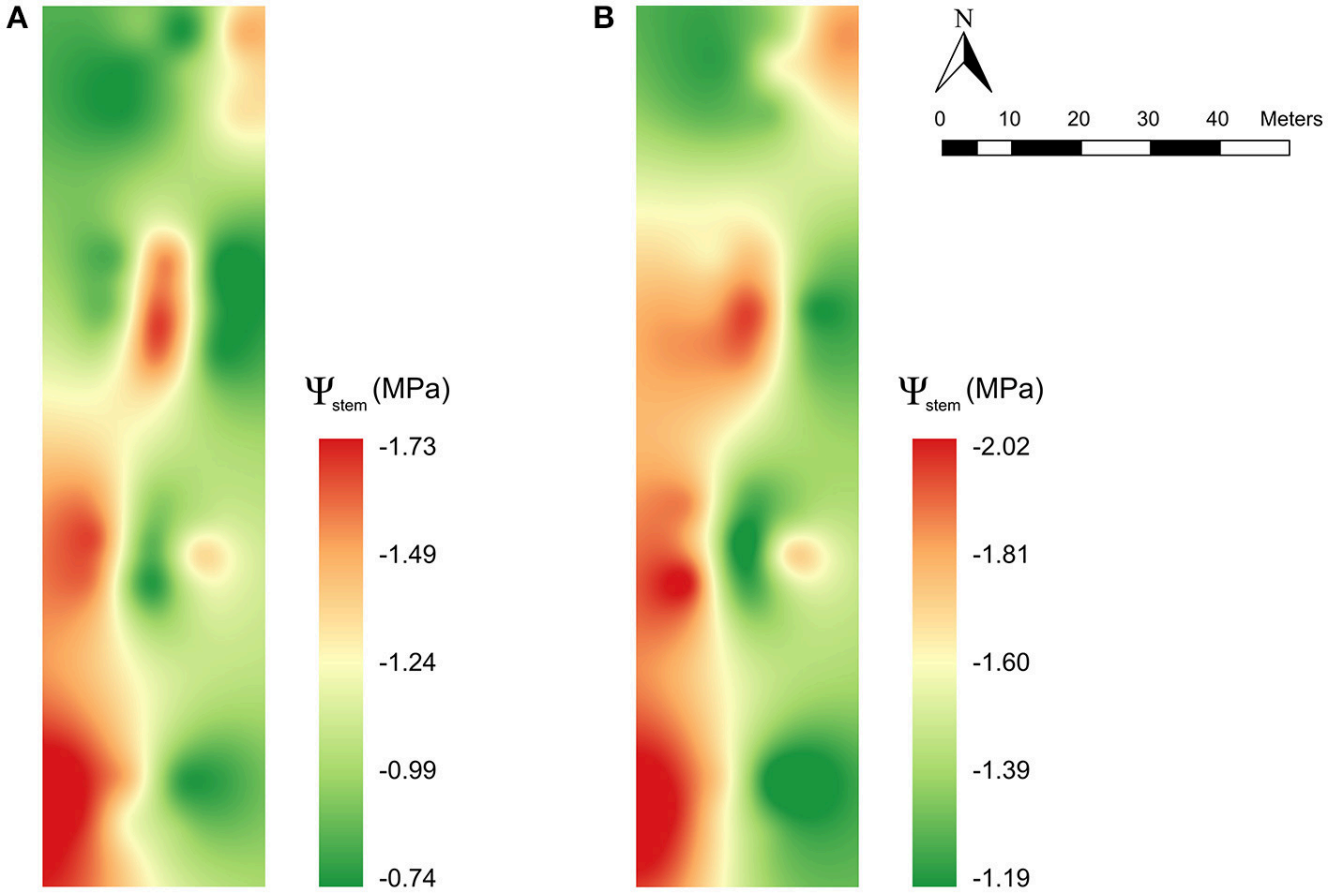
Maps of the spatial variability of the plant water status using the predicted values of stem water potential (ψ_stem_) obtained from the models built from NIR spectra acquired on-the-go at 5 km/h on the east side of the canopy on the **(A)** 11th September 2015 and **(B)** 23rd August 2016.

The spatial variability of the vineyard water status at two given dates of season 2015 (Figure 8A) and 2016 (Figure 8B) was computed and presented as maps from the predicted values of ψ_s_ obtained using the external prediction models from the NIR spectra acquired on-the-go. The most stressed vines (with more negative ψ_s_ values) were found on the west side of the plot and toward the north east, while the plants in the east and north west parts of the plot exhibited little to no water stress.

## Discussion

A novel, non-destructive methodology based on NIR spectroscopy acquired on-the-go, to assess the vineyard water status has been developed and validated over two seasons. The presented results evidence the capability of on-the-go proximal NIR spectroscopy to successfully determine the grapevine water status in a commercial vineyard, using robust and reliable prediction models for the quantification of midday stem water potential (ψ_s_), which is a widely-used plant water status indicator (Choné et al., 2001). The outcomes obtained from a comprehensive internal (cross validation) and external validation (prediction) of the method over several dates, from pre-veraison to harvest over two seasons, with substantial differences in the environmental conditions of air temperature, relative humidity, and vapor pressure deficit, confirm the robustness and soundness of the developed on-the-go NIR spectroscopy method for plant water status assessment.

The physical principle underlying the interaction between the electromagnetic radiation at the NIR wavelength used in the present work (1,100–2,100 nm) and the grapevine canopy leaves, calls for a predominant absorbance by the OH group of water, which constitutes between 80 and 90% of living cells (Williams, 2000). Likewise, the spectral response by the water molecules of plant leaves to NIR radiation in the range between 1000 and 2500 nm has motivated previous studies in which plant water status was successfully assessed from NIR spectroscopy acquired manually with portable devices in several crops (Santos and Kaye, 2009; De Bei et al., 2011; Vila et al., 2011; Poblete-Echeverría et al., 2014; Gutiérrez et al., 2016; Tardaguila et al., 2017). While the outcomes obtained in these studies were satisfactory and encouraging, manual methods are time consuming and labor demanding, therefore unsuitable for the acquisition of many measurements within a limited time frame in a vineyard plot, necessary to characterize the spatial variability of vineyard water status. One step forward was recently taken by Diago et al. (2017), who estimated the stomatal conductance (g_s_) of grapevines using a NIR spectrophotometer mounted on an all-terrain-vehicle that acquired spectral measurements at 25 and 50 cm from the canopy while the vehicle was stopped, facing the targeted vine. In the present work, not only was spectral acquisition carried out contactless, at 30 cm from the canopy, but also on-the-go, from a vehicle moving at a speed commonly used by agricultural vehicles in vineyard operations. This advance toward automated, on-the-go monitoring of the plant water status features a real capability of vineyard water status variability assessment, with a performance in terms of ${\text{R}}_{\text{p}}^{2}$ and sensitivity (prediction RMSE) similar or superior to the manual (Santos and Kaye, 2009; De Bei et al., 2011; Vila et al., 2011; Gutiérrez et al., 2016; Tardaguila et al., 2017) and stop-and-go approaches (Diago et al., 2017).

The sensitivity of the on-the-go NIR spectroscopy in-field monitoring to predict the ψ_s_ ranged from 0.131 to ~0.190 MPa, regardless the scale. Compared to other technologies, such as thermography, aimed at non-destructively assessing the plant water status, the sensitivity of the presented on-the-go NIR spectroscopy is similar to that provided by aerial or manual thermal imaging (García-Tejero et al., 2016). However, a remarkable benefit of on-the-go NIR spectroscopy over thermography is the lack of temperature references, such as T_dry_ and T_wet_ (Jones, 1999) needed to compute the most widely used thermal indices, such as the crop water stress index (CWSI) (Idso et al., 1981) and the Jones index (Ig) (Jones et al., 2002) More specifically, over aerial thermography, the advantages of proximal, on-the-go NIR spectroscopy also include the measurement of the lateral side of the canopy instead of the zenithal view (Baluja et al., 2012), a higher temporal flexibility for revisiting (measurement of a given vineyard at several dates across the season), the larger operational times (no batteries of limited power are used), and the absence of legal issues.

In vineyards planted with north-south row orientation, such as the one of this study, the spectral information procured from the east side (morning side) of the canopy generated more accurate predictions of ψ_s_ than the spectra corresponding to the west side. This was evidenced by the higher values of ${\text{R}}_{\text{cv}}^{2}$ and ${\text{R}}_{\text{p}}^{2}$ and smaller values of CV-RMSE and prediction RMSE of the east-derived models. The choice of row orientation in vineyard establishment has a strong impact on the environmental conditions of the two canopy sides, especially in terms of sun radiation. The influence of sun radiation in leaf structural features (Hanson, 1917) is well-recognized, and the effect of leaf structural changes in its physiological response (Lo Gullo and Salleo, 1988) and reflectance values in the NIR range between 750 and 1,350 nm against water constraints is also demonstrated (Slaton et al., 2001). The wavelength range used in the present study (1,100–2,100 nm) partly includes the NIR range sensitive to differences in leaf structure. Therefore, differences in leaf structural properties between leaves from the east and west sides of a grapevine canopy may exist, and the acquired NIR spectra may have perceived them. As a result, the models built from east and west side spectra exhibited differential performances in predicting ψ_s._ Nevertheless, the models generated from spectra acquired on the two sides of the canopy also yielded very satisfactory results. From a practical point of view, this would imply the possibility of taking spectral measurements either from the east or west side of the vineyard rows, depending on the direction in which the vehicle moves along the rows, and the frequency of measurements (e.g., every other row, one row every four rows, etc.).

Several factors borne in mind in the validation of the present method account for its robustness. These include: two spatial scales (field replication and sub replication unit), two cross validation approaches (10-fold venetian blinds and leave one day out cross validation), and the inclusion of a large number of data taken at different dates under changing environmental conditions. With regard to the spatial scale, on-the-go NIR spectroscopy has proved to successfully assess the plant water status either at a 6 m (sub replication unit) or 30 m (field replication) long sections of vineyard row in the studied plot. This “spatial resolution” may vary across vineyard plots depending on their soil variability and topography. However, the rate of acquisition of the spectral system (24 Hz, that is 24 spectra per second) is high enough to provide about 18 spectra per row meter (e.g., it can be assimilated to one vine, depending on the inter row spacing), provided the all-terrain vehicle moves at 5 km/h. Of the two cross validation approaches, the leave one day out cross validation was selected and tested by its practical implications as it mimics the estimation of the plant water status values of a day of measurements using a model built previously with data from other dates. Therefore, in a real situation, the assessment of the water status within a vineyard at a given date, using the developed on-the-go NIR spectroscopy could be done following the leave one day out cross validation approach (in which no reference data at the day of interest is needed) or pursuing the external prediction approach, for which some reference data (ψ_s_) of a limited number of control points have to be taken to feedback the model. The performance of this second option is superior to the leave one day out cross validation approach, especially in terms of sensitivity (0.0546 MPa on average), but the lack of any reference sampling in the latter may counterbalance the drop in accuracy (lower R^2^ and higher SE). Differences between the two approaches may be shortened or enlarged depending upon the magnitude of variation in the environmental conditions between the unknown date and those used to build the model. In this context the third factor emerges. In the present study, nine dates with substantial differences in air T, RH, and VPD were included to develop and validate the model. This ensures its robustness and prevents overfitting, which is the limitation to proper usage of the predictive model in days with very similar environmental conditions to those of the dates involved in model development. For a given location, an initially built model could become more robust by including some reference data from different days and seasons on a dynamic basis (model feedback). Similarly, models can be either site-specific or involve different plots; either variety-specific or multi-variety. The choice for one option or another is equally valid, provided a good calibration, encompassing representative samples of the potential variability to account for that of the unknown population is conducted.

The capability of on-the-go NIR spectroscopy to assess the grapevine water status and the large number of measurements this technique can provide in a flexible and reliable way, enables the quantification and mapping of the variability of the plant water status of a vineyard. As observed in the maps for two given dates in seasons 2015 and 2016, two differentiated zones of plant water status within the studied plot could be delineated (green vs. other than green areas in the maps of this work) and separately irrigated using different watering doses and schedules. Hence, these vineyard water status maps obtained from the NIR-based predicted values of ψ_s_ are very valuable for the wine industry, in terms of sustainability and cost savings (e.g., Water and energy saving) particularly in the current woldwide scenario of increased water scarcity.

Although the on-the-go NIR spectroscopy proximal method has proven to successfully monitor the spatial variability of the grapevine water status within a vineyard, further research involving a wider range of grapevine cultivars, seasons, and locations should be conducted with the goal of developing even more accurate and robust, global predictive models. Another approach to be tested would involve the identification of the most discriminant wavelengths in the spectral range of operation in the built models. This would open a new line of research that could potentially enable the definition and computation of spectral indices in this NIR region, which respond to the plant water status changes.

## Conclusion

A helpful tool to assess the plant water status in vineyards involving proximal NIR spectroscopy, acquired on-the-go from a moving vehicle has been generated and extensively validated against the stem water potential, which is a widely used plant water status indicator. The developed models proved to be robust and capable of yield water status estimations in commercial vineyards. Their performance and the vast amount of data that this on-the-go spectral solution provides, facilitates the exploitation of this non-destructive technology to appraise and map the vineyard water status variability with a high spatial and temporal resolution. This spectral system could be installed in any agricultural vehicle and its measurements would be of great interest and help to the grape and wine industry to define and schedule precise irrigation strategies.

## Author contributions

MD and JT: Designed the experimental layout, helped during field measurements and wrote the manuscript; JF-N and SG: Acquired spectral measurements, prepared some figures and built the maps; SG: Created the algorithm for spectral assignment; JF-N and MM: Analyzed the data and built the models.

### Conflict of interest statement

The authors declare that the research was conducted in the absence of any commercial or financial relationships that could be construed as a potential conflict of interest.

## References

[B1] Acevedo-Opazo C. Ortega-Farias S. Fuentes S. (2010). Effects of grapevine (*Vitis vinifera* L.) water status on water consumption, vegetative growth and grape quality: an irrigation scheduling application to achieve regulated deficit irrigation. Agric. Water Manage. 97, 956–964. 10.1016/j.agwat.2010.01.025

[B2] Acevedo-Opazo C. Tisseyre B. Guillaume S. Ojeda H. (2008). The potential of high spatial resolution information to define within-vineyard zones related to vine water status. Precis. Agric. 9, 285–302. 10.1007/s11119-008-9073-1

[B3] Ballester C. Buesa I. Bonet L. Intrigliolo D. S. (2014). Usefulness of stem dendrometers as continuous indicator of loquat trees water status. Agric. Water Manag. 142, 110–114. 10.1016/j.agwat.2014.04.019

[B4] Baluja J. Diago M. P. Balda P. Zorer R. Meggio F. Morales F. (2012). Assessment of vineyard water status variability by thermal and multispectral imagery using an unmanned aerial vehicle (UAV). Irrig. Sci. 30, 511–522. 10.1007/s00271-012-0382-9

[B5] Barnes R. J. Dhanoa M. S. Lister S. J. (1989). Standard normal variate transformation and de-trending of near-infrared diffuse reflectance spectra. Appl. Spectrosc. 43, 772–777. 10.1366/0003702894202201

[B6] Bellvert J. Marsal J. Girona J. Gonzalez-Dugo V. Fereres E. Ustin S. L. (2016). Airborne thermal imagery to detect the seasonal evolution of crop water status in peach, nectarine and Saturn peach orchards. Remote Sens. 8:39 10.3390/rs8010039

[B7] Brereton R. G. (2003). Principal component analysis: the method, in Chemometrics. Data Analysis for the Laboratory and Chemical Plant, ed Brereton R. G. (Chichester: John Wiley and Sons, Ltd.), 191–223.

[B8] Chapman D. M. Roby G. Ebeler S. E. Guinard J. X. Matthews M. A. (2005). Sensory attributes of cabernet sauvignon wines made from vines with different water status. Austr. J. Grape Wine Res. 11, 339–347. 10.1111/j.1755-0238.2005.tb00033.x

[B9] Chaves M. M. Santos T. P. Souza C. R. D. Ortu-o M. F. Rodrigues M. L. Lopes C. M. (2007). Deficit irrigation in grapevine improves water-use efficiency while controlling vigour and production quality. Ann. Appl. Biol. 150, 237–252. 10.1111/j.1744-7348.2006.00123.x

[B10] Choné X. Van Leeuwen C. Dubourdieu D. Gaudillère J. P. (2001). Stem water potential is a sensitive indicator of grapevine water status. Ann. Bot. 87, 477–483. 10.1006/anbo.2000.1361

[B11] Cohen Y. Alchanatis V. Saranga Y. Rosenberg O. Sela E. Bosak A. (2017). Mapping water status based on aerial thermal imagery: comparison of methodologies for upscaling from a single leaf to commercial fields. Precis. Agric. 5, 801–822. 10.1007/s11119-016-9484-3

[B12] Cozzolino D. (2014). An overview of the use of infrared spectroscopy and chemometrics in authenticity and traceability of cereals. Food Res. Int. 60, 262–265. 10.1016/j.foodres.2013.08.034

[B13] Cozzolino D. Cynkar W. U. Shah N. Smith P. (2011). Multivariate data analysis applied to spectroscopy: potential application to juice and fruit quality. Food Res. Int. 44, 1888–1896. 10.1016/j.foodres.2011.01.041

[B14] Dambergs R. Gishen M. Cozzolino D. (2015). A review of the state of the art, limitations, and perspectives of infrared spectroscopy for the analysis of wine grapes, must, and grapevine tissue. Appl. Spectrosc. Rev. 50, 261–278. 10.1080/05704928.2014.966380

[B15] De Bei R. Cozzolino D. Sullivan W. Cynkar W. Fuentes S. Dambergs R. (2011). Non-destructive measurement of grapevine water potential using near infrared spectroscopy. Aust. J. Grape Wine Res. 17, 62–71. 10.1111/j.1755-0238.2010.00117.x

[B16] Dhanoa M. S. Lister S. J. Barnes R. J. (1995). On the scales associated with near-infrared reflectance difference spectra. Appl. Spectrosc. 49, 765–772. 10.1366/0003702953964615

[B17] Diago M. P. Bellincontro A. Scheidweiler M. Tardaguila J. Tittmann S. Stoll M. (2017). Future opportunities of proximal near infrared sensing approaches to determine vine water. Aust. J. Grape Wine Res. 23, 409–414. 10.1111/ajgw.12283

[B18] Fernández J. E. (2014). Plant-based sensing to monitor water stress: applicability to commercial orchards. Agric. Water Manag. 142, 99–109. 10.1016/j.agwat.2014.04.017

[B19] Fernández J. E. Cuevas M. V. (2010). Irrigation scheduling from stem diameter variations: a review. Agric. For. Meteorol. 150, 135–151. 10.1016/j.agrformet.2009.11.006

[B20] Fernández-Novales J. Tardaguila J. Gutiérrez S. Marañón M. Diago M. P. (2018). In field quantification and discrimination of different vineyard water regimes by on-the-go NIR spectroscopy. Biosyst. Eng. 165, 47–58. 10.1016/j.biosystemseng.2017.08.018

[B21] Fuentes S. De Bei R. Pech J. Tyerman S. (2012). Computational water stress indices obtained from thermal image analysis of grapevine canopies. Irrig. Sci. 30, 523–536. 10.1007/s00271-012-0375-8

[B22] García-Tejero I. F. Costa J. M. Egipto R. Durán-Zuazo V. H. Lima R. S. N. Lopes C. M. (2016). Thermal data to monitor crop-water status in irrigated Mediterranean viticulture. Agric. Water Manag. 176, 80–90. 10.1016/j.agwat.2016.05.008

[B23] Geladi P. Manley M. Lestander T. (2003). Scatter plotting in multivariate data analysis. J. Chemometr. 17, 503–511. 10.1002/cem.814

[B24] Gutiérrez S. Diago M. P. Fernández-Novales J. Tardaguila J. (2017). On-the-go thermal imaging for water status assessment in commercial vineyards, in Advances in Animal Biosciences Precision Agriculture, Vol. 8, (Edinburgh, UK: ECPA), 520–524.

[B25] Gutiérrez S. Tardaguila J. Fernández-Novales J. Diago M. P. (2016). Data mining and NIR spectroscopy in viticulture: applications for plant phenotyping under field conditions. Sensors 16:236. 10.3390/s16020236 26891304 PMC4801612

[B26] Hanson H. C. (1917). Leaf-structure as related to environment. Am. J. Bot. 4, 533–560. 10.2307/2435253

[B27] Hinkelmann K. Kempthorne O. (2007). Randomized Block Designsm, in Design and Analysis of Experiments, 2nd Edn (Hoboken, NJ: John Wiley & Sons, Inc.), 277–372. Available online at: http://onlinelibrary.wiley.com/doi/10.1002/9780470191750.ch6/summary

[B28] Hotelling H. (1931). A generalisation of student's ratio. Ann. Math. Stat. 2, 360–378. 10.1214/aoms/1177732979

[B29] Idso S. B. Jackson R. D. Pinter P. J. Reginato R. J. Hatfield J. L. (1981). Normalizing the stress-degree-day parameter for environmental variability. Agric. Meteorol. 24, 45–55. 10.1016/0002-1571(81)90032-7

[B30] Jackson E. J. (2003). A User's Guide to Principal Components. New York, NY: John Wiley & Sons, Inc.

[B31] Jones H. G. (1999). Use of infrared thermometry for estimation of stomatal conductance as a possible aid to irrigation scheduling. Agric. For. Meteorol. 95, 139–149. 10.1016/S0168-1923(99)00030-1

[B32] Jones H. G. (2004). Irrigation scheduling: advantages and pitfalls of plant-based methods. J. Exp. Bot. 55, 2427–2436. 10.1093/jxb/erh213 15286143

[B33] Jones H. G. Grant O. M. (2016). Remote sensing and other imaging technologies to monitor grapevine performance, in Grapevine in a Changing Environment: A Molecular and Ecophysiological Perspective, eds Gerós H. Chaves M. M. Medrano H. Delrot S. (Chichester: Wiley-Blackwell), 179–196.

[B34] Jones H. G. Stoll M. Santos T. De Sousa C. Chaves M. M. Grant O. M. (2002). Use of infrared thermography for monitoring stomatal closure in the field: application to grapevine. J. Exp. Bot. 53, 2249–2260. 10.1093/jxb/erf083 12379792

[B35] Lo Gullo M. A. Salleo S. (1988). Different strategies of drought resistance in three Mediterranean sclerophyllous trees growing in the same environmental conditions. New Phytol. 108, 267–276. 10.1111/j.1469-8137.1988.tb04162.x 33873932

[B36] Nicolaï B. M. Beullens K. Bobelyn E. Peirs A. Saeys W. Theron K. I. (2007). Nondestructive measurement of fruit and vegetable quality by means of NIR spectroscopy: a review. Postharvest Biol. Technol. 46, 99–118. 10.1016/j.postharvbio.2007.06.024

[B37] Ojeda H. Andary C. Kraeva E. Carbonneau A. Deloire A. (2002). Influence of pre-and postveraison water deficit on synthesis and concentration of skin phenolic compounds during berry growth of *Vitis vinifera* cv. Shiraz. Am. J. Enol. Vit. 53, 261–267.

[B38] Poblete-Echeverría C. Ortega-Farías S. Lobos G. A. Romero S. Ahumada L. Escobar A. (2014). Non-invasive method to monitor plant water potential of an olive orchard using visible and near infrared spectroscopy analysis. Acta Hortic. 1057, 363–368. 10.17660/ActaHortic.2014.1057.43

[B39] Rodriguez-Dominguez C. M. Ehrenberger W. Sann C. Rüger S. Sukhorukov V. Martín-Palomo M.-J. (2012). Concomitant measurements of stem sap flow and leaf turgor pressure in olive trees using the leaf patch clamp pressure probe. Agric. Water Manag. 114, 50–58. 10.1016/j.agwat.2012.07.007

[B40] Santos A. O. Kaye O. (2009). Grapevine leaf water potential based upon near infrared spectroscopy. Sci. Agric. 66, 287–292. 10.1590/S0103-90162009000300001

[B41] Savitzky A. Golay M. J. E. (1964). Smoothing and Differentiation of data by simplified least squares procedures. Anal. Chem. 36, 1627–1639. 10.1021/ac60214a047

[B42] Slaton M. R. Hunt E. R. Jr. Smith W. K. (2001). Estimating near-infrared leaf reflectance from leaf structural characteristics. Am. J. Bot. 88, 278–284. 10.2307/2657019 11222250

[B43] Tardaguila J. Fernández-Novales J. Gutiérrez S. Diago M. P. (2017). Non-destructive assessment of grapevine water status in the field using a portable NIR spectrophotometer. J. Sci. Food Agric. 97, 3772–3780. 10.1002/jsfa.8241 28133743

[B44] Van Leeuwen C. Tregoat O. Choné X. Bois B. Pernet D. Gaudillére J.-P. (2009). Vine water status is a key factor in grape ripening and vintage quality for red bordeaux wine. How can it be assessed for vineyard management purposes? J. Int. Sci. Vigne Vin. 43, 121–134. 10.20870/oeno-one.2009.43.3.798

[B45] Vila H. Hugalde I. Di Filippo M. (2011). Estimation of leaf water potential by thermographic and spectral measurements in grapevine. RIA 37, 46–52.

[B46] Williams L. E. (2000). Grapevine water relations, in Raisin Production Manual ed Christensen L. P. (Oakland, CA: University of California), 121–126.

[B47] Williams P. Norris K. (2001). Near-Infrared Technology in the Agricultural and Food Industries. St. Paul, MN: American Association of Cereal Chemists.

[B48] Wold S. Sjöström M. Eriksson L. (2001). PLS-regression: a basic tool of chemometrics. Chemometr. Intell. Lab. Syst. 58, 109–130. 10.1016/S0169-7439(01)00155-1

